# Data quality in an HIV vaccine efficacy clinical trial in South Africa: through natural disasters and with discipline

**DOI:** 10.1186/s12874-023-01967-9

**Published:** 2023-06-24

**Authors:** Fatima Laher, Mookho Malahleha, Shelly Ramirez, William Brumskine, Kennedy Otwombe, Zoe Moodie, Mary Allen

**Affiliations:** 1 Perinatal HIV Research Unit, Faculty of Health Sciences, University of the Witwatersrand, Diepkloof, P.O. Box 114, Johannesburg, 1864 Soweto South Africa; 2 Synergy Biomed Research Institute, East London, Eastern Cape South Africa; 3 Vaccine and Infectious Disease Division, Fred Hutchinson Cancer Center, Seattle, Washington USA; 4 The Aurum Institute NPC, Johannesburg, Gauteng South Africa; 5 Department of Medicine, School of Medicine, Vanderbilt University, Nashville, TN USA; 6 School of Public Health, Faculty of Health Sciences, University of the Witwatersrand, Johannesburg, South Africa; 7 Vaccine Research Program, Division of AIDS, National Institute of Allergy and Infectious Diseases, National Institutes of Health, Bethesda, MD USA

**Keywords:** Protocol deviation, Preventive action, Clinical trial, Data quality, Improvement science

## Abstract

**Background:**

To produce quality data that informs valid clinical trial results and withstands regulatory inspection, trial sites should adhere to many complex and dynamic requirements. Understanding non-conformance to requirements informs the emerging field of improvement science. We describe protocol deviations in South Africa’s largest HIV vaccine efficacy trial.

**Methods:**

We analysed data from the HVTN 702 trial using mixed methods. We obtained descriptive statistics, from protocol deviation case report forms collected from 2016–2022, of deviation by participant, trial site, and time to site awareness. We thematically analysed text narratives of deviation descriptions, corrective and preventive actions, generating categories, codes and themes which emerged from the data.

**Results:**

For 5407 enrollments, 4074 protocol deviations were reported (75 [95% CI: 73.0–77.6] deviations per 100 enrolments). There was a median of 1 protocol deviation per participant (IQR 1–2). Median time from deviation to site awareness was 31 days (IQR 0–146). The most common category of deviation type was omitted data and/or procedures (69%), and 54% of these omissions were stated to have arisen because of the national lockdown at the beginning of the COVID-19 pandemic. The ratio of protocol deviations to cumulative enrolments was highest in the year 2020 (0.34). Major themes of deviations were: COVID-19 and climate disasters giving rise to deviation trends, subroutines introducing an opportunity for deviation, and document fragmentation (such as requirements dispersed across multiple guidance documents) as an obstacle. Preventive action categories were: no preventive measures; discipline, training and/or awareness; quality review, checking and verifying and changing the process and/or implementation tools. Major themes of preventive actions were that systems-based actions are unusual, with people-based actions dominating, and that root cause analysis was rarely mentioned.

**Conclusions:**

In the age of infectious and climate disaster risks, trials may benefit from simple study designs and trial-related documents. To optimise protocol adherence, sponsors and sites should consider ongoing training, and routinely review deviation reports with a view to adjusting processes. These data quality lessons may inform future trial design, training and implementation.

**Trial registration:**

HVTN 702 was registered with the South African National Clinical Trials Register (DOH-27–0916-5327) and ClinicalTrials.gov (NCT02968849).

**Supplementary Information:**

The online version contains supplementary material available at 10.1186/s12874-023-01967-9.

## Background

To obtain valid results of a clinical trial, trial sites should adhere to the principles of Good Clinical Practice, good documentation practices, site operating procedures and protocol-related guidance when collecting and reporting data. The concept of data quality in clinical research is multidimensional, including best practices in data collection, adherence to protocol requirements, adherence to regulatory and ethical requirements, metrics (reporting timelines, query rates, query resolution) and mitigating against and preventing data errors that may adversely impact research outcomes or trial participant safety. This multiplicity of requirements is complex to implement. Common quality issues in clinical trials include missing data, incorrect data, inconsistencies in data collection, and delayed data reporting [1]. High rates of missed procedures, errors, and missing or inaccurate data, especially for items related to the endpoints of trial objectives, have the potential to jeopardize participant safety, and impede the ability of researchers to answer the scientific questions of the trial [1]. Therefore, checking data quality and addressing arising issues throughout a trial is a requirement of Good Clinical Practice [2].

Maintaining good data quality is also important to withstand inspection by regulatory agencies, especially because some agencies control product licensure for market. A small proportion of trial sites are inspected by regulators. In 13 Good Clinical Practice inspections of clinical trial sites in Africa, the European Medicines Agency identified 7 critical findings at 4 sites, related to data management, handling of investigational product and protocol compliance [3]. Similarly, in clinical trial site inspections conducted by the Food and Drug Administration (FDA) of the United States of America, violations were related to procedures, consent process, investigational product and study records. Inspection findings are rarely disclosed in research publications [4, 5].

A clinical trial is a massive undertaking of effort and financial resources. Understanding non-conformance to requirements, in the intricate and dynamic context of site implementation, offers an opportunity to contribute to the emerging field of improvement science for clinical trials [6]. We aimed to describe protocol deviations in a large trial in South Africa by a mixed methods investigation: quantitative and qualitative analyses. Our purpose was to enrich knowledge of issues which could potentially affect data quality and to document lessons learned.

## Methods

### Summary of trial complexity

To date, the HIV Vaccine Trials Network (HVTN) 702 trial is the HIV vaccine efficacy trial which enrolled the most number of participants in South Africa [7]. It was a randomised, double-blind, phase 2b/3 placebo-controlled trial conducted at 14 sites supported by 7 peripheral blood mononuclear cell processing laboratories. The trial compared the safety and efficacy of an ALVAC/gp120 + MF59 vaccine regimen to placebo [8].

During the development of the first protocol version, rapid process improvement workshops were held, using lean thinking concepts to reduce redundancies and optimise trial processes and tools. There were 4 versions of the protocol, released in the years 2015, 2016, 2017 and 2020, as well as 108 bulletins, 10 clarification memos, 5 communiques, and 21 memos. Trial sites were trained in person or online before new protocol versions were implemented.

Participants were required to provide handwritten informed consent. There were multiple procedures, including evaluation of 14 inclusion criteria based on general and demographic factors, HIV status, laboratory inclusion values and reproductive status. There were 20 exclusion criteria based on general factors, vaccines and other injections, immune system factors, and clinically significant medical conditions. HIV-uninfected participants completed up to 21 different types of clinical procedures, including vaccination at 5–6 timepoints, and up to 20 different types of laboratory procedures, depending on the protocol version, and there was a maximum of 18 scheduled visits over three years. Participants who acquired HIV infection underwent up to 8 clinical and 12 laboratory procedures.

Data were collected on paper first, and then captured into imedidata, an electronic data capture system. Data quality performance metrics (data entry timelines, adverse event entry timelines, query rates, query resolution timelines) were shared with sites continuously in real time.

The national regulator conducted Good Clinical Practice inspections at some trial sites in 2017 (the Medicines Control Council of South Africa inspected 5 trial sites) and in 2020 (the South African Health Products Regulatory Agency inspected 1 trial site). The sites passed all inspections.

The interim data safety monitoring board meeting in January 2020 declared non-efficacy. Thereafter, vaccinations were stopped, participants were unblinded, and post-unblinding safety visits were continued until close-out in 2021.

### Ethical approvals

Approval was obtained from the research ethics committees of the University of the Witwatersrand, University of Cape Town, University of KwaZulu-Natal, Sefako Makgatho University, and the South African Medical Research Council. Research was performed in accordance with the relevant guidelines and regulations and in accordance with the Declaration of Helsinki. HVTN 702 was registered with the South African National Clinical Trials Register (DOH-27–0916-5327 on 12 Jan 2016) and ClinicalTrials.gov (NCT02968849 on 21 Nov 2016).

### Trial non-conformance procedures

Site staff members, monitors and site liaison managers were required to inform the Investigator of Record of any reportable non-conformance incidents. Reportable protocol deviations included any incidents or omissions in study conduct, regardless of whether they added risk to the participant; and non-adherence to the protocol, study documents, International Conference on Harmonisation E6 (R1) Guideline for Good Clinical Practice, FDA guidelines, and local regulatory requirements.

HVTN 702 site staff reported protocol deviations in a protocol deviation case report form captured into imedidata. Protocol deviation data were reviewed weekly by the HVTN 702 protocol safety review team, and when necessary were referred to the DAIDS Office of Clinical Site Oversight. These data were also used to compile non-conformance listings for submission to regulatory agencies. To help preserve blinding of other study staff, potential accidental unblinding incidents were not reported through the case report form but rather to a clinical trial manager.

All trial sites were required to have quality management plans in place. The HVTN appointed site liaision managers to help trial sites assure quality and to advise on quality improvement. The sponsor appointed a clinical research organisation to conduct monitoring regularly at sites.

### Measures

We obtained data entry metrics, cumulatively to March 2022, from the HVTN 702 data quality management report: the number of case report form pages entered, the number of adverse events entered, the number of data queries, and the number of responded queries.

We analysed HVTN 702 protocol deviation case report forms which had been entered into the electronic database by 16 March 2022, collating the assigned participant identification number, trial site, date of trial site awareness, deviation date, deviation type, deviation description, corrective action and preventive action. A variable was created for time to site awareness, which subtracted the date of site awareness from the deviation date.

We collated the total number of enrolments, dates of enrolment, and date of final visit of all HVTN 702 participants.

### Quantitative analyses

We calculated descriptive statistics, including frequencies, using Microsoft Excel and SAS Enterprise Guide 7.15 (SAS Institute Inc., Cary, NC, USA). Categories of protocol deviations and preventive actions were summed. Median and interquartile ranges (IQR) were determined for continuous measures overall and by groupings. Sites were divided into high and low enrolment categories using the median number of enrolments as a cut-off (Supplementary Fig. 1). Deviation rates and their 95% confidence intervals were estimated.

### Qualitative analyses

We applied thematic analysis to the text narratives of the deviation description, steps taken to address the deviation, and steps taken to prevent future occurrences of the deviation. These narratives had been written by trial site staff and entered into the protocol deviation case report form for each protocol deviation. When performing thematic analysis, we conducted the steps of immersion, coding, categorising and generation of themes [9]. The analyst read through all text narratives to gain immersion into the data, then generated categories from the data. The categories were shared with and validated by another author who had been reading the data weekly during the trial. Using Microsoft Excel, the analyst assigned categories to each protocol deviation narrative; then created, refined and assigned codes which emerged from the data. The analyst then generated major and minor themes which emerged from the data. Quotations from the narratives were chosen to exemplify the themes and add contextual knowledge. Possibly identifying data were removed to maintain confidentiality.

## Results

### Data entry metrics

During the trial, 447,130 case report forms were entered into the database, and 94% were entered within 7 days of the visit date. There were 8716 adverse events entered, and 85% were entered within 3 days of the date that the adverse event was reported to the trial site. The data query ratio was 5 per 100 case report form pages, and 75% of query responses were within 7 days of the visit date (Table 1).

**Table 1:** Metrics of data entry into electronic database during the HVTN 702 trial for all 14 trial sites (cumulative to March 2022)

Case report form pages entered within 7 days of visit/all expected pages (%)	Adverse events entered within 3 days of reporting to trial site/all adverse events entered (%)	Queries/pages entered (%)	Query response within 7 days of query opening/queries answered (%)
421427/447130 (94%)	7424/8716 (85%)	37741/781460 (5%)	28436/37735 (75%)

### Protocol deviations

There were a total of 5407 participants enrolled, amongst which 4074 protocol deviations were reported over 12,456.1 person years of follow-up, corresponding to a protocol deviation rate of 32.7 [95% CI: 31.7–33.7] per 100 person years. The ratio of deviations to enrolments (4074/5407) was 75 (95% CI: 73.0–77.6) per 100 enrolments. At least one protocol deviation was reported for 2687 participants. There was a median of 1 protocol deviation per participant (IQR 1–2).

The median time from deviation to site awareness was 31 days (IQR 0–146). The fourteen sites had a median of 275 deviations per site (IQR 116–425) (Table 2).

**Table 2:** Protocol deviations by year and site

	**Number of protocol deviations, (%**^**a**^**) (*N* = 4074)**	**Cumulative number of enrollments (*N* = 5407)**	**Ratio of protocol deviations to cumulative enrollments (95% CI)**
**Year**			
**2016**	16 (0.39%)	54	0.30 (0.18–0.48)
**2017**	1221 (29.97%)	2335	0.52 (0.49–0.55)
**2018**	723 (17.75%)	4614	0.16 (0.15–0.17)
**2019**	237 (5.82%)	5407	0.04 (0.039–0.05)
**2020**	1815 (44.55%)	5407	0.34 (0.32–0.35)
**2021**	62 (1.52%)	5407	0.01 (0.009–0.014)
**Site**			
**A**	237 (5.82%)	505	0.47 (0.41–0.53)
**B**	116 (2.85%)	429	0.27 (0.23–0.32)
**C**	100 (2.45%)	492	0.20 (0.17–0.25)
**D**	313 (7.68%)	511	0.61 (0.55–0.69)
**E**	363 (8.91%)	354	1.03 (0.93–1.14)
**F**	478 (11.73%)	353	1.35 (1.24–1.48)
**G**	367 (9.01%)	407	0.90 (0.81–1.0)
**H**	425 (10.43%)	333	1.28 (1.16–1.40)
**I**	86 (2.11%)	64	1.34 (1.09–1.66)
**J**	661 (16.22%)	358	1.85 (1.71–1.99)
**K**	87 (2.13%)	483	0.18 (0.15–0.22)
**L**	117 (2.87%)	486	0.24 (0.20–0.29)
**M**	189 (4.64%)	342	0.55 (0.48–0.64)
**N**	535 (13.13%)	290	1.85 (1.70–2.0)
**All sites**	4074 (median 275, IQR 116–425)	5407 (median 382.5, IQR 342–486)	1.33 (median 0.76, IQR (0.27–1.34)

^a^Percentages may not total 100 due to rounding

Deviation type categories were omitted data and/or procedures (69%), error in data or procedures (13%), use of materials not approved by the research ethics committee (10%), consent errors (4%), and needless data collection or procedures (3%) (Table 3).

**Table 3:** Categories of 4074 reported protocol deviations

Deviation categories	N (%^a^)	Requirement not met (may overlap and be ≤ or ≥ N)
**Omitted data or procedures**	2815 (69.10%)	Omissions were reported on laboratory tests (*n* = 1902), physical examinations (*n* = 1604), eligibility (*n* = 338), adverse event reporting (*n* = 119), questionnaires (*n* = 226), counselling (*n* = 63), full visits (*n* = 57), non-study product treatment provision (*n* = 36), study product (*n* = 13), and regulatory approvals (*n* = 9).
**Error in data or procedures**	545 (13.38%)	There were errors on physical examination (*n* = 263), laboratory tests (*n* = 103), adverse events and reactogenicity (*n* = 52), study product (*n* = 51), participant unique identifier assignment, eligibility, enrolment and randomisation (*n* = 29), delays with implementing updated study materials (*n* = 15), the full visit (*n* = 8), reimbursement (*n* = 4), non-study treatment (*n* = 3), questionnaires (*n* = 3), and counselling (*n* = 1). The major errors with physical examinations were related to thermometry methodology inconsistent with protocol. Regarding laboratory tests, a prominent issue was not following requirements to take some samples before certain events.
**Use of materials not approved by ethics committee**	406 (9.97%)	Study materials distributed to participants before ethics committee approval were study brochures (*n* = 403) and participant diary cards (*n* = 3).
**Consent errors**	172 (4.22%)	Consent errors were implementing the incorrect consent form version (*n* = 112), documentation issues (*n* = 39), delays in implementing an approved consent form (*n* = 18) and 2 could not be coded. Documentation issues included form completion that did not meet good documentation practice standards. It also included consent process documentation that was insufficiently detailed, e.g. the distribution of copies, or whether a participant understood explanations of their incorrect responses on their assessments of understanding consent.
**Needless data collection or procedures**	130 (3.19%)	Unnecessary data collection or procedures were laboratory samples (*n* = 113), full visits (*n* = 7), questionnaires (*n* = 4), study product (*n* = 1), treatment (*n* = 1), and unblinding (*n* = 1).
**Not categorised**	6 (0.15%)	Insufficient information for categorization (*n* = 6).

^a^Percentages may not total 100 due to rounding

At each site, a median number of 382.5 (IQR 342–486) participants was enrolled (Table 2). Enrolments at high-enrolling sites ranged from 407 to 511 participants and from 64 to 358 participants at low-enrolling sites, with equal numbers of sites in each category. The protocol deviation to enrolment ratio was 0.48 at high-enrolling sites (1598/3313) versus 1.18 at low-enrolling sites (2476/2094). Low-enrolling sites had a higher proportion of omitted data and procedures and use of materials not approved by the Ethics Committee compared to high-enrolling sites. However, there was a higher proportion of errors, consent documentation errors and needless data and procedures at high-enrolling sites compared to low-enrolling sites (Table 4, Supplementary Table 1).

**Table 4:** Protocol deviations by site enrolment

Protocol deviation category	Total protocol deviations (*N* = 4074), (%^a^)	Protocol deviations at high enrolling sites (*N* = 1598), (%^a^)	Protocol deviations at low enrolling sites (*N* = 2476), (%^a^)
Error in data/procedures	545 (13.38%)	316 (19.77%)	229 (9.25%)
Consent documentation errors	172 (4.22%)	147 (9.20%)	25 (1.01%)
Needless data collection/procedures	130 (3.19%)	72 (4.51%)	58 (2.34%)
Omitted data/procedure	2815 (69.10%)	1058 (66.21%)	1757 (70.96%)
Data for categorisation insufficient	6 (0.15%)	2 (0.13%)	4 (0.16%)
Use of materials not approved by Ethics Committee	406 (9.97%)	3 (0.19%)	403 (16.28%)

^a^Percentages may not total 100 due to rounding

From qualitative analyses, using quantitative methods as supportive evidence, four main deviations themes are discussed.

### Deviation theme 1: disasters can give rise to protocol deviation trends

The national response to COVID-19 began soon after trial unblinding, affecting trial conduct. Of omitted data in the trial, 54% (1525/2815) was stated to be because the South African government imposed lockdown at the beginning of the COVID-19 pandemic. This was linked with omissions of physical examinations and laboratory samples, while procedures like questionnaires, counselling, and event reporting were conducted telephonically.

*“The participant missed the following visit 08.0 assessment due to the conduct of a telephonic visit: vital signs assessment, collection of blood, pregnancy and STI samples. The telephonic visit was conducted as a result of the COVID-19 lockdown period in the country.”*

There was also record of a climate disaster, flooding, linked with protocol deviations. One site reported that their city *“experienced extreme weather conditions, resulting in a cancellation of the full meeting”* of their ethics committee. The ethics committee therefore could not provide continuing review, and the annual recertification lapsed over six days when the trial site conducted study procedures with nine participants.

### Deviation theme 2: subroutines introduce an opportunity for deviation

The concept of a “subroutine” is borrowed from the field of computer programming, where it means a sequence of programming instructions for a specific scenario. The concept is similar to an algorithm in clinical care – “if this happened, then do that”. In clinical trials, subroutine errors occur when staff do not follow the dissimilar sequence of procedures required for different participants or scenarios.

In HVTN 702, many omissions were linked with not following subroutines. A prominent example of deviations related to a multi-subroutine was stool swab collection. The main informed consent form provided participants with the option to collect swabs of their stool, which initiated the first subroutine for staff to either collect or not collect the samples. The second subroutine was that staff should ask only participants who collected the stool swab to complete a dietary and gastrointestinal symptom questionnaire. The third subroutine was the timing of the swab sample: staff should collect the stool swab before study product administration at the month 0 visit, and any time during the month 6.5 visit. There were protocol deviations reported for each subroutine, e.g. collecting stool samples from participants who had not agreed to it on their consent form, not completing the required questionnaires when stool samples were completed, and collecting samples after study product administration at the month 0 visit.

*“The research nurse erroneously collected stool samples and participant did not consent for stool sample collection on the signed informed consent form...”*

Protocol deviations reported for some other subroutines included not following subroutines specified in the study specific procedures guide for anaemic participants, omitting to collect dried blood spot samples for participants whose visits were on specific calendar days as specified in the study specific procedures guide or collecting it at the wrong time during the visit, and not tailoring sample types for sexually transmitted infection tests by sex at birth.

*“Dried blood spot specimen taken after vaccination.”*

### Deviation theme 3: document fragmentation presents obstacles for consent documentation

More common than implementing incorrect consent form versions was the prevalence of giving participants study-related brochures before its approval by the ethics committee. The template informed consent form in the protocol made provision for four issues to be covered in more depth in brochures: trial-permitted birth control methods, trial procedures, vaccine-induced seroposititvity (VISP), and participant bill of rights and responsibilities. Site investigators could submit these as separate documents to their ethics committees for approval to distribute to participants with the consent form, or copy the information from 3 of these brochures (with the exception of vaccine-induced serposititvity information) into their site consent form in lieu of brochures.

Distributing consent-related brochures to participants before ethics committee approval was common to 403 deviations.

*“Participant was handed with VISP and Bill of rights brochures at screening before they were reviewed and approved by ethics committee.”*

Furthermore, deviations were reported when the structure of a consent document fragmented the completion of fields across multiple pages. Deviations reported that staff and participants omitted to complete any fields situated on pages other than the signature page of the consent form.

*“Pharmacogenetic ICF [VERSION, DATE] page 3 of 4 no option was chosen by participant regarding limited pharmacogenetic testing in error. Participant continued the study without confirming if they still want [to] allow use of samples for limited pharmacogenetic testing or not.”*

### Deviation theme 4: visit scheduling

Not checking the visit window when scheduling participants and/or before conducting procedures was linked with visit procedures being performed unnecessarily.

*“Pregnancy test performed out of window as a result of incorrect visit scheduling.”*

In many of deviations reported for missed visits, calls had not been made to remind participants of their upcoming visits. In some cases, staff had not recognised that the visit window was open for a participant who was at the site presenting for ancillary care, and did not take the opportunity to do visit procedures. In other cases, a visit was split over more than one day, and participants did not return for the second part of the split visit.

### Preventive actions

Preventive action categories were: (i) no preventive measures (*n* = 1903); (ii) discipline, training and/or awareness (*n* = 963); (iii) quality review, checking and verifying (*n* = 839), and (iv) changing the process and/or tools (*n* = 633). In 254 cases, more than one category of preventive action had been applied.

### Preventive action theme 1: systems-based actions are unusual

Some narratives did not provide anything further than corrective action. There was also evidence from the narratives that some staff were unclear about the difference between corrective and preventive actions. In an example of a laboratory sample not collected from the participant, the narrative for the corrective action described the efforts undertaken to obtain the sample after deviation awareness, and the preventive action narrative describes the corrective action further:

*“Participant visit window for visit 13.0 is still open. Closes on the 11 may 2019. CT/NG [Chlamydia trachomatis, Neisseria gonorrhoeae] urine will be collected at this visit when the participant is found. CT/NG urine kits are available.”*

Some narratives proposed that the preventive action would be to stop the incorrect action and simply follow the correct action in future. Specific plans on how the site would change course were not always evident in the narratives. In an example of missed counselling and questionnaires, the narrative stated the site’s steps to prevent future occurrences of this type of deviation:

*“Staff to always follow visit schedule and do procedures according to visit requirements.”*

Instead, many narratives offered preventive actions centred around people, and there was a dominant perception that an error would prompt people to change behaviors, become more *“vigilant”* and exercise more care. The onus was placed on people to remember to improve their performance after training and awareness campaigns through email reminders or wallcharts.

*“Staff member will avoid being distracted by the participant's conversation and pay more attention to visit procedures…”*

*“Staff member responsible was made aware of the error and the importance of following the schedule of procedures as stipulated in Appendix H of the Protocol was emphasised. ‘Appendix H’ Page was printed, laminated and placed in the counselling rooms for easy reference.”*

For a haemoglobin test that could not be done because of a clotted sample:

*“Nurses reminded of the gentle inversion technique to prevent clotting.”*

Systems-based preventive actions were less usual. They focused on correcting or adding more detail to forms (e.g. laboratory requisition forms, medical history forms), adding more detail to checklists to distil visit requirements (e.g. visit flow checklists), introducing trackers (e.g. for informed consent versions), adding staff members to perform critical requirements which were not being done (e.g. assigning staff members to call participants to remind them of their scheduled visits, allocating staff members to call participants for safety follow up), and procuring tools with advanced technology (e.g. imaging technology that locates veins for successful blood sampling to prevent insufficient blood sampling, and syncronising digital clocks to a device with atomic time instead of digital clocks to prevent time documentation discrepancies).

In 2% (*n* = 79) of the 4074 protocol deviation narratives, site staff mentioned that they had conducted root cause analysis of that particular deviation. Staff often narrated that they found a human root cause – i.e. they thought that the error arose from a person.

## Discussion

The application of considerable rigor is required for the successful implementation of large clinical trials aiming to maintain high data quality meeting regulatory agency standards. With a median of 1 protocol deviation per participant, our study results show that, in a large complex trial in South Africa, a high level of data quality was achievable. Deviations were most common in the beginning of the trial, decreasing thereafter, suggesting that trial sites have a “learning curve”. High-enrolling trial sites had fewer errors per enrolled participant than lower-enrolling sites, a finding that possibly counters the intuition that busy sites make more errors. Our data do not explain the observation. Perhaps sites which enroll more participants reach quality proficiency faster; perhaps sites with more resources can enroll more participants and have more resources for quality procedures; or perhaps sites which make fewer errors can spend less time on the re-work of addressing errors which allows them to devote more time to enrolling more participants.

Currently, we are not aware of standardized mathematically-based metrics for trial quality. We would not consider the whole number of deviations to be entirely useful. Different trials have different ‘opportunities’ (e.g. number of enrolments, data points, visits) for deviation. Compared to a small trial, the quality of larger trials may therefore be less affected by a specific number of deviations. A denominator allows us the ability to make comparisons. Although current standardized trial reporting guidelines do not require reporting a quality metric and thus we cannot compare across trials, here we report a ratio of 75 deviations per 100 enrolments.

A major theme in our study was that of disasters – COVID-19 and climate – giving rise to a substantial proportion of deviations: over half of all data omissions were stated to be because of lockdown. This suggests an emerging need for increased flexibility in future study design and implementation. Conventionally, trials were designed so that data were collected at trial sites. In the present day, emerging infectious diseases and climate disasters are identified as major disruptive risks [10]. Disaster management and mitigation plans may aid trial sites. Future setting-specific research should investigate the outcomes of trial decentralization in low and middle income countries. Examples of trial decentralization innovations include electronic consenting, and data collection through telephonic visits, home visits, and internet visits such as video calls and participant apps.

A second prominent theme was the interplay between complexity and error. Making a study procedure requirement contingent upon another factor introduced opportunities for deviation. Simple trial designs, which minimise subroutines and instead standardise as many procedures as possible for all trial participants, may achieve better data quality. Decreasing document fragmentation may make protocol adherence easier: e.g. locating all requirements for a procedure in one document instead of dispersing over protocols, manuals, and various communications. For informed consent forms, the authors recommend including all information inside one leaflet instead of referring to multiple documents, and also placing all fields for completion on a single page. Other authors have suggested that, in order to optimise data quality, trials should minimise the amount of data collected to only those most necessary to answering the objectives [11]. Those authors highlight that beyond the requirement to collect, process, and review the quality, there are also cost and time implications to each data item. Sponsors may consider if providing suggested and well-designed standardised data collection tools would be useful. Last, trial sites should implement document version control processes.

Our analysis highlights sponsor responsibility in overseeing quality management. Especially in complex multicentre trials, sponsors often contract monitoring functions to other organisations. In this case, the sponsor additionally reviewed deviations and ensured that sites were enacting quality measures. In addition, the HVTN provided support personnel at the site to help sites find and resolve issues. With this infrastructure in place, the median time to detection of issues was about a month, sufficiently early to implement corrective and preventive actions.

The distinction between corrective and preventive actions is not only semantic [12]. Corrective actions try to remedy the specific error being reported, and in doing so, permit the opportunity for data quality to improve for that specific participant and data point. Preventive actions, however, afford the opportunity to investigate the system creating the problem, proactively improve processes, and avoid similar future incidents. In our analysis, trial sites were unable to formulate preventive actions for about half of all deviations: many of those deviations were because of COVID-19 and climate disasters and therefore beyond investigator control. When preventive actions were taken, people-based actions were common: asking staff to be more disciplined, training and re-training staff and/or raising awareness about the deviation and the correct way of conduct. Preventive actions which focused on improving processes or tools were the least common.

Root-cause analysis is not a preventive action in itself, but has been described as a systematic approach to uncover causal factors of an issue, for a more informed guidance of preventive actions [13]. Despite multiple methodologies having been defined, root cause analysis is known to be challenging to conduct. In the narratives studied here, root cause analysis was rarely mentioned, and when it was, deficiencies in processes or tools were not stated. Instead implications were largely on the people who made the deviation. Although we are unaware of data proving the efficacy of root cause analysis as a tool used to inform improvement of trial site processes, it has been shown to be useful in other contexts [14]. It would be helpful for future studies to investigate the efficacy of root causes analysis in reducing protocol deviations in clinical trials.

A possible limitation of our analysis is missed or inaccurate identification and/or reporting of protocol deviations. Missed reporting may have resulted in underestimation of protocol deviations. Inaccurate reporting may have resulted in overestimation or underestimation of categories of protocol deviations. The likelihood of reporting issues was minimized by different groups reviewing data quality: site staff, HVTN site liason teams, a contract research organization appointed by the sponsor, and the database managers.

Thematic analysis allowed us to analyse a large amount of text but its main limitation is researcher bias: our understanding based on our perceptions and experiences may have led us to infer meanings from the narratives.

The use of both qualitative and quantitative methods is a strength of this analysis. The nature of data collection through narratives (instead of questions with standardized pre-defined answer options that allow statistical analysis) allowed us to develop specific insights into the circumstances around deviations, providing us with a richer understanding than one can obtain from quantitative methodology alone. For example, although root cause analysis was rarely mentioned in the narratives of preventive action, this does not mean it was not performed in the cases when it was not mentioned.

## Conclusions

This analysis offers many data quality lessons of practical value. These lessons may be helpful not only for the HIV vaccine field – which faces a future of more trials in order to discover a highly effective product – but also to help prepare and train for trials of other products [7].

First, in the age of infectious and climate disaster risks, trials may benefit from designs which build in flexible options for data collection continuity.

Second, routine real-time provision of deviation reports may help the sponsor and protocol leadership teams to assist site investigators to adapt rapidly to critical arising issues, and allocate attention and resources accordingly. Multi-level quality review during the trial may contribute to process and quality improvement measures.

Third, to address site staff turnover and adaptation to evolving protocol requirements, staff training is a need that is ongoing throughout the trial to minimize protocol non-adherence.

Last, protocol deviations may offer a helpful opportunity to site investigators to review processes. When trial sites can access their data quality metrics in real time, they can focus efforts appropriately.

## Supplementary Information

**Additional file 1: Supplementary Table 1.** Distribution of categories of deviations (*N*=4074) across the 14 trial sites. **Supplementary Figure 1.** Box and whisker plot of number of participants enrolled by high- and low-enrolling sites.

## Data Availability

The data that support the findings of this study are available on request from the corresponding author. Data is currently not available to the public due to publication restrictions.

## Abbreviations

COVID-19: Coronavirus Disease 2019
CT/NG: Chlamydia trachomatis and Neisseria gonorrhoeae
FDA: Food and Drug Administration
HIV: Human Immunodeficiency Virus
HVTN: HIV Vaccine Trial Network

## References

[1] Friedman LM, Furberg CD, DeMets DL, Reboussin DM, Granger CB. Fundamentals of Clinical Trials. 5th ed. e-book: Springer, 2015, pp.233–250.

[2] International Conference on Harmonisation. Efficacy guidelines. https://www.ich.org/page/efficacy-guidelines (Accessed 01 Jul 2022).

[3] European Medicines Agency. Classification and analysis of the GCP inspection findings of GCP inspections conducted at the request of the CHMP. https://www.ema.europa.eu/en/documents/other/classification-analysis-good-clinical-practice-gcp-inspection-findings-gcp-inspections-conducted_en.pdf (Accessed 01 Jul 2022).

[4] Garmendia CA, Epnere K, Bhansali N (2018). Research deviations in FDA-regulated clinical trials: a cross-sectional analysis of FDA inspection citations. Ther Innov Regul Sci.

[5] Seife C (2015). Research misconduct identified by the US Food and Drug Administration: out of sight, out of mind, out of the peer-reviewed literature. JAMA Intern Med.

[6] Girdler SJ, Glezos CD, Link TM (2016). The science of quality improvement. JBJS Rev.

[7] Laher F, Bekker LG, Garrett N (2020). Review of preventative HIV vaccine clinical trials in South Africa. Arch of Virol.

[8] Gray GE, Bekker LG, Laher F (2021). Vaccine efficacy of ALVAC-HIV and Bivalent Subtype C gp120-MF59 in adults. N Engl J Med.

[9] Green J, Willis K, Hughes E (2007). Generating best evidence from qualitative research: the role of data analysis. Aust N Z J Public Health.

[10] Lawrence JM, Ibne Hossain NU, Jaradat R (2020). Leveraging a Bayesian network approach to model and analyze supplier vulnerability to severe weather risk: A case study of the U.S. pharmaceutical supply chain following Hurricane Maria. Int J Disaster Risk Reduct.

[11] O’Leary E, Seow H, Julian J (2013). Data collection in cancer clinical trials: too much of a good thing. Clin Trials.

[12] Motschman TL, Moore SB (1999). Corrective and preventive action. Transfus Sci.

[13] Spath P (1998). Add root-cause analysis to your PI armament. Hosp Peer Rev.

[14] Shah F, Falconer EA, Cimiotti JP. Does root cause analysis improve patient safety? A systematic review at the Department of Veterans Affairs. Qual Manag Health Care Epub ahead of print. 2022. 10.1097/QMH.0000000000000344. 10.1097/QMH.0000000000000344 35170581

