# Supplementary material for: Data quality in an HIV vaccine efficacy clinical trial in South Africa: through natural disasters and with discipline
Source: BMC Med Res Methodol. 2023 Jun 24;23:147. doi: 10.1186/s12874-023-01967-9 (PMC10290289; doi:10.1186/s12874-023-01967-9)
Supplement: Supplementary file 1 — Additional file 1: Supplementary Table 1. Distribution of categories of deviations (N=4074) across the 14 trial sites. Supplementary Figure 1. Box and whisker plot of number of participants enrolled by high- and low-enrolling sites. [file 12874_2023_1967_MOESM1_ESM.docx]

**Supplementary Table 1: Distribution of categories of deviations (N=4074) across the 14 trial sites**

| **Trial Site** | **Omitted data or procedures (%)** | **Error in data or procedures (%)** | **Use of materials not approved by Ethics Committee (%)** | **Consent errors (%)** | **Needless data collection or procedures (%)** | **Not categorised, (%)** |
| --- | --- | --- | --- | --- | --- | --- |
| A | 69 (29.11) | 38 (16.03) | 0 (0.00) | 123 (51.90) | 6 (2.53) | 1 (0.42) |
| B | 52 (44.83) | 51 (43.97) | 0 (0.00) | 11 (9.48) | 2 (1.72) | 0 (0.00) |
| C | 62 (62.00) | 25 (25.00) | 0 (0.00) | 1 (1.00) | 11 (11.00) | 1 (1.00) |
| D | 277 (88.50) | 16 (5.11) | 0 (0.00) | 0 (0.00) | 20 (6.39) | 0 (0.00) |
| E | 337 (92.84) | 15 (4.13) | 0 (0.00) | 2 (0.55) | 9 (2.48) | 0 (0.00) |
| F | 462 (96.65) | 13 (2.72) | 0 (0.00) | 1 (0.21) | 2 (0.42) | 0 (0.00) |
| G | 194 (52.86) | 154 (41.96) | 3 (0.82) | 4 (1.09) | 12 (3.27) | 0 (0.00) |
| H | 15 (3.53) | 6 (1.41) | 403 (94.82) | 0 (0.00) | 1 (0.24) | 0 (0.00) |
| I | 44 (51.16) | 14 (16.28) | 0 (0.00) | 7 (8.14) | 21 (24.42) | 0 (0.00) |
| J | 560 (84.72) | 82 (12.41) | 0 (0.00) | 9 (1.36) | 7 (1.06) | 3 (0.45) |
| K | 55 (63.22) | 14 (16.09) | 0 (0.00) | 6 (6.90) | 11 (12.64) | 1 (1.15) |
| L | 76 (64.96) | 28 (23.93) | 0 (0.00) | 1 (0.85) | 12 (10.26) | 0 (0.00) |
| M | 100 (52.91) | 81 (42.86) | 0 (0.00) | 3 (1.59) | 5 (2.65) | 0 (0.00) |
| N | 512 (95.70) | 8 (1.50) | 0 (0.00) | 4 (0.75) | 11 (2.06) | 0 (0.00) |
| **Total** | **2815 (69.10)** | **545 (13.38)** | **406 (9.97)** | **172 (4.22)** | **130 (3.19)** | **6 (0.15)** |


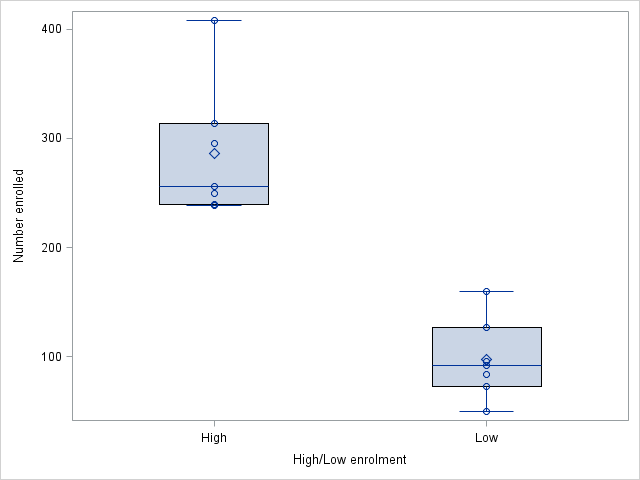


**Supplementary Figure 1: Box and whisker plot of number of participants enrolled by high- and low-enrolling sites.**
